# Quantitative Residual Stress Analysis in Steel Structures Using EMAT Nonlinear Acoustics

**DOI:** 10.3390/s25227019

**Published:** 2025-11-17

**Authors:** Kaleeswaran Balasubramaniam, Borja Nuevo Ortiz, Álvaro Pallarés Bejarano

**Affiliations:** Innerspec Technologies, Calle Sanglas 13, 28890 Madrid, Spainapallares@innerspec.com (Á.P.B.)

**Keywords:** residual stress, EMAT, nonlinear ultrasonics, XRD, coercivity, nondestructive testing

## Abstract

Residual stress plays a critical role in the durability and structural integrity of steel rolls and bars. Proper analysis helps prevent defects like warping or cracking, ensuring the steel meets quality standards and performs reliably in critical applications. This paper presents a methodology for analysing residual stresses using electromagnetic acoustic transducer (EMAT) based nonlinear ultrasonics. It compares its effectiveness with established techniques such as X-ray diffraction (XRD) and coercive force measurements. The results demonstrate that nonlinear ultrasonics provides more detailed insights into stress distribution, particularly in subsurface regions where traditional methods like XRD face limitations. It also shows good sensitivity to stress-induced microstructural variations than coercive force measurements. This research study is the first to perform a comparative analysis using XRD, EMAT, and coercive force techniques on industrial samples, followed by the implementation of EMAT nonlinear technology at an industrial production site. The findings indicate a positive trend observed in XRD and coercive force results, and those from nonlinear ultrasonics, further validating its accuracy. Moreover, the technology has been successfully applied in steel manufacturing industries through the project named STEEL components assessment using a novel non-destructive residual stress ultrasonic technology (STEELAR), funded by the Research Fund for Coal and Steel (RFCS). These findings underscore the potential of nonlinear ultrasonics as a powerful, fast and complementary tool for comprehensive residual stress monitoring in steel components, enhancing both theoretical understanding and practical industrial application.

## 1. Introduction

Residual stresses within steel components can significantly impact their mechanical properties, performance, and longevity. Accurate analysis of these stresses [1] is essential for ensuring the reliability of steel rolls and bars used in various industrial applications. Traditional methods like X-ray diffraction (XRD) and coercive force measurements have been instrumental in assessing residual stresses. However, these techniques often face limitations in detecting subsurface stresses and capturing detailed microstructural changes, particularly in regions below the surface where XRD typically focuses. Nonlinear ultrasonics offers a promising alternative by probing deeper into the material and providing insights into subsurface residual stresses and associated microstructural variations at greater depths, which conventional non-destructive testing (NDT) methods cannot visualise. Unlike traditional methods, nonlinear ultrasonics can detect subtle stress-induced changes that are sometimes missed by XRD and coercivity measurements.

Various researchers worked on nonlinear ultrasonics to check the residual stress in structures. Ultrasonic nonlinear analysis in steel plates was conducted by Jiao et al. using a collinear Lamb wave mixing technique [2], which analysed residual stress measurements. A finite element modelling (FEM) study paper by Jiang et al. simulated stress distributions in multilayer composites [3], showcasing the efficacy of acoustoelastic effects in detecting stress variations across different layers. Sampath et al. explored the application of nonlinear ultrasonics for depth profiling [4], demonstrating how this method can effectively map residual stress distributions in surface-treated metallic structures. The paper by Geimer et al. introduces white-noise excitation as a novel approach for in situ stress analysis [5], highlighting the role of nonlinear parameters in enhancing the detection of stress variations.

Kim et al. developed theoretical insights and practical applications of nonlinear ultrasonic phenomena [6], providing a foundation for understanding material behaviour under stress and during microstructural changes. Gao et al. [7] developed an analytical model linking acoustic nonlinearity to dislocation characteristics, which is crucial for understanding the microstructural impact on ultrasonic wave propagation. Song et al. [8] discuss high-energy ultrasonic methods used for both the detection and regulation of residual stress in aluminium alloys, emphasizing the dual functionality of these techniques. Mao et al. in [9] investigate the use of critically refracted longitudinal waves for steady-state stress evaluation, showcasing their ability to provide accurate stress measurements under constant loading conditions. Dunn et al. [10] highlighted the effectiveness of vibro-ultrasonic techniques in detecting damage within composite laminates, particularly in regions with pre-existing stress concentrations.

Kim et al. explored the sensitivity of nonlinear ultrasonic methods [11] to microstructural changes due to second-phase precipitation, which is crucial for monitoring ageing and degradation processes. Kim et al. studied ultrasonic nonlinearity in response to changes in prestress [12] within the concrete, offering an NDT method to evaluate structural integrity. Zhang et al. in [13] evaluated the feasibility of using nonlinear properties of critically refracted waves for residual stress detection, presenting a novel approach for NDT. Yee et al. proposed cross-correlation filtering in [14] to improve nonlinear ultrasonic measurements during fatigue testing, which is critical for monitoring material degradation. Yan et al. investigated the relationship between stress and nonlinear ultrasonic coefficients in 2024 aluminium [15], providing insights into stress monitoring in aerospace applications.

Nilsson et al. studied corrosion [16] in steel structures using nonlinear ultrasonics and tried to correlate the nonlinear parameters with corrosion degradation. Yan et al. developed a method of exciting reversed-phase Rayleigh waves in opposite directions [17] and identified a remarkable increase in the acoustic non-linearity parameter. The research paper [18] uses a noncontact technique using air-coupled transducers for detecting fatigue cracks in rotating steel shafts. By generating ultrasonic waves at two distinct frequencies, the presence of a crack is identified through nonlinear modulation. The research paper in [19] studied the nonlinear guided wave propagation in prestressed aluminium plates associated with the second harmonic generation by using 2D FEM simulations. The model employed is a second-order approximation accounting for material and geometric nonlinearities. The best-identified electromagnetic acoustic transducer (EMAT) paper [20] on nonlinear ultrasonics uses only the second harmonics and the birefringence approach as a monitoring parameter and correlates cuts in the structures with harmonics. The very recent publication [21] uses the birefringence theory [22] for monitoring residual stresses in rail wheels.

Each paper contributes to advancing ultrasonic testing techniques for residual stress analysis, impacting material evaluation, structural health monitoring and industrial applications. By integrating theoretical models, advanced signal processing and practical testing, these studies highlight the potential of nonlinear ultrasonics to improve the accuracy and efficiency of residual stress measurements across various materials. However, several drawbacks remain:

Firstly, despite its potential, EMAT technology [23] has not been extensively utilized or compared with methods like XRD or coercivity measurements in residual stress analysis. There is an opportunity to fully explore EMAT, particularly in non-contact and hard-to-reach environments, and to conduct comparative studies with established techniques.

Secondly, many EMAT studies, such as the one referenced in [20], focus narrowly on second harmonics in plates and some focus on the birefringence [23] theory-based solutions (pointwise residual stress analysis). This limits the understanding of EMAT’s broader capabilities in capturing complex stress distributions and microstructural changes. Future research could benefit from exploring additional nonlinear parameters or higher-order harmonics to gain deeper insights into material behaviour under stress.

Thirdly, there is a lack of comprehensive studies comparing nonlinear ultrasonic techniques across various materials and stress conditions. Such comparative research could identify the most effective methods for specific industrial applications, especially in steel manufacturing, where precise residual stress monitoring is crucial. Finally, while some papers concentrate on theoretical modelling or practical applications of nonlinear ultrasonics, there is limited integration between these approaches. Bridging this gap could lead to more robust models that better predict real-world stress distributions, thus improving residual stress measurement accuracy. Most of the proposed methods were time-consuming and were mostly pointwise applications, meaning they were not used for a continuous monitoring process using the NDT technology.

Addressing existing gaps in residual stress analysis presents a significant opportunity to advance the understanding, application, and comparison of nonlinear ultrasonic techniques, especially in critical industries. This research paper is particularly noteworthy as it emphasizes the use of nonlinear ultrasonics generated from transmitting Rayleigh waves [24] through an EMAT device, for early-stage residual stress monitoring in larger industrial steel structures. To the authors’ knowledge, this study is the first to apply EMAT-based nonlinear ultrasonics for monitoring large industrial steel structures using continuous assessment and to perform a comparative pointwise analysis of industrial samples with traditional NDT methods, such as XRD and coercive force analysis, against the EMAT nonlinear method.

The research demonstrates a positive trend between β (non-dimensional parameter) and XRD between the nonlinear ultrasonic methods and conventional techniques, underscoring the potential of nonlinear ultrasonics for testing larger steel structures. By offering a comparative evaluation, the study highlights the advantages of nonlinear ultrasonics in terms of sensitivity and depth of analysis. This approach not only enhances the understanding of stress distributions within steel components but also provides a more comprehensive view of their structural integrity. The findings from this research emphasize the significant potential of nonlinear ultrasonics as a valuable tool for residual stress analysis. The insights gained could lead to improvements in material performance and extend the lifespan of steel components in industrial settings.

## 2. Nonlinear Ultrasonics

The acoustoelastic effect [25] is a useful technique for determining stress within solid materials. This method is based on the fundamental principle that the application of stress or mechanical load alters the propagation of ultrasonic waves through the material. One of the most noticeable consequences of the acoustoelastic effect, particularly in isotropic solid materials, is the emergence of acoustic anisotropy under stress. This phenomenon results in the formation of two distinct shear modes, each with its own velocity and polarization relative to the direction of the applied stress.

The two stress-induced shear modes have different velocities. The difference in shear wave velocities causes a phenomenon known as birefringence [22] theory. Birefringence is the splitting of a single incident ultrasound wave into two separate waves, each moving at its own speed. The relative shear-wave velocity difference, denoted as ‘a’ and expressed as ∆V/V, is an important parameter in this context. It is critical for quantifying the extent of stress-induced anisotropy while also accounting for any inherent material anisotropy. Equation (1) is used to calculate absolute stress (σ) in a material:

(1) σ=K×(a)

where ‘K’ is the acoustoelastic constant. This constant, ‘K,’ is important in stress calculations and can be determined only experimentally. The formula for ‘K’ is 8μ^2^/(4μ + n), where ‘μ’ signifies the material’s shear modulus, and ‘n’ corresponds to Murnaghan’s third-order elastic modulus. It is worth noting that ‘n’ reflects the material’s non-linear behaviour arising from stress.

**Figure 1:**
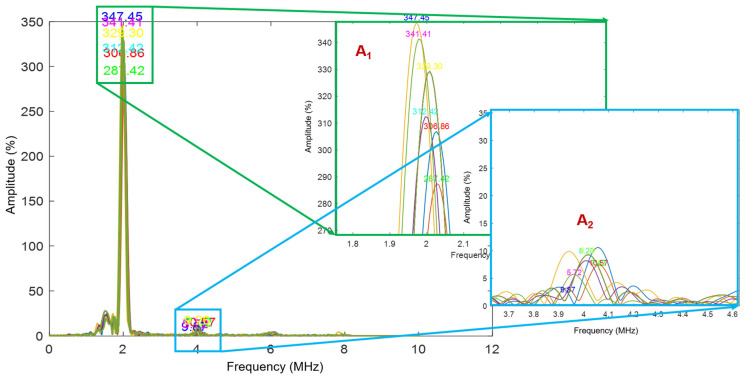
Exemplary FFT of Rayleigh wave group signals with 2nd harmonics obtained using EMAT after converting it from the specific time cut domain.

The presence of residual stress within the samples introduces non-linearity into the system, leading to the generation of higher harmonics in the Rayleigh waves, as depicted in the exemplary Figure 1 (A_2_). In nonlinear ultrasonic signal processing, a single-frequency ultrasonic wave (A_1_) is sent into a material. In a perfectly elastic (linear) medium, the received signal contains only this fundamental frequency. However, when the material has stresses or microstructural defects such as microcracks, dislocations, or voids, it behaves nonlinearly. This nonlinearity causes part of the energy from the fundamental wave to transfer into higher harmonics, primarily the second harmonic (A_2_) at twice the original frequency. By analyzing the amplitudes of A_1_ and A_2_ typically through the Fourier transform and computing their ratio (A_2_/A_1_^2^), the level of material nonlinearity can be quantified.

In cases when the material under investigation is extremely thick or elongated, traditional stress measurement methods based on bulk shear waves may be ineffective. In such cases, researchers consider Rayleigh waves as an alternative. To quantify this non-linearity and its relationship to residual stress [15], the study employed Equation (2):

(2) $$\beta \propto \frac{A_{2}}{A_{1}^{2}}$$

In this equation, ‘β’ (beta) represents the non-linearity parameter (a non-dimensional value), and it is directly proportional to the relative residual stress present in the material. A_1_ is the fundamental frequency (1st harmonics), and A_2_ is the second harmonics (as shown in Figure 1). The parameter is determined by measuring both the amplitude of the main lobe and the harmonics within the Fast Fourier Transform (FFT) [24] of the Rayleigh wave signals, which are obtained by taking the main signal of the obtained Rayleigh waves in the time domain and converting it to the frequency domain. More details about the time-frequency conversion are shown in Section 4.2.1. This approach allows for continuous relative residual stress level monitoring in the examined materials.

## 3. Specimens: Laboratory and Industrial

**Figure 2:**
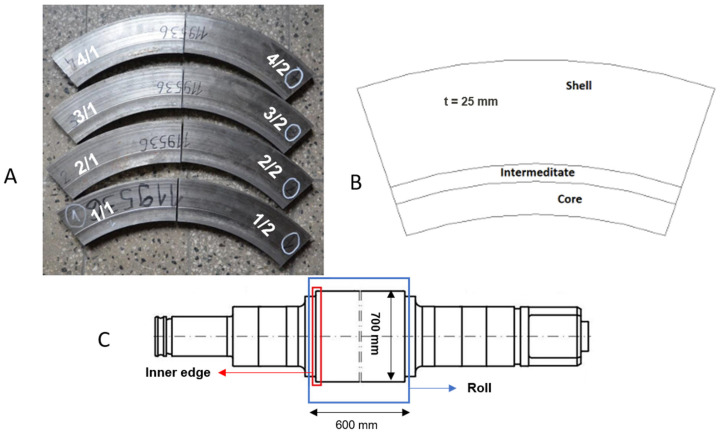
(**A**) Cutout delivered samples, (**B**) composition and shape of the delivered samples, (**C**) position of the ring cut out from the roll.

The roll ring samples provided by Valji d.o.o, Slovenia, were cut into four segments in the shape of a ring (Figure 2A) prepared from the rolled shell as shown in Figure 2B with a Young’s modulus (E) value of 210 GPa and Poisson’s ratio (ν) of 0.29. The segments were cut from the surface of the roll and contained the working layer (Figure 2C), the whole transition or intermediate layer, and a small portion of the core material. The ring was cut out before the roll was heated to introduce different levels of residual stress after laboratory heat treatment. According to the information from the supplier of testing pieces, the roll’s heat treatment consists of austenitization at 950 °C and subsequent tempering at 550 °C.

To develop the requested different levels of internal (residual) stress, three cooling regimes of the delivered segments were chosen: quenching into the water, quenching into oil and air cooling. The different heat treatments produce distinct microstructures (e.g., variations in phase, grain size, and dislocation density), which affect the material’s elastic nonlinearity. As a result, the nonlinear ultrasonic signals reflect not only residual stresses but also microstructural differences.

The three delivered samples were divided into two halves by the spark discharge method (EDM) to get pairs of samples in the as-quenched and tempered state. The EDM process minimizes the mechanical and thermal effects. This process causes negligible stress relaxation, and measurements were taken at the central regions, well away from cut edges. Therefore, the prestress field in the specimens was not significantly affected by the removal process. The last segment (segment No. 1/1, 1/2—marked in Figure 2A) was left in the as-delivered state and served as the reference material. The heat-treatment parameters for the individual segments listed in Table 1 are provided by Materials and Metallurgical Research sro (MMV) in the Czech Republic.

**Figure 3:**
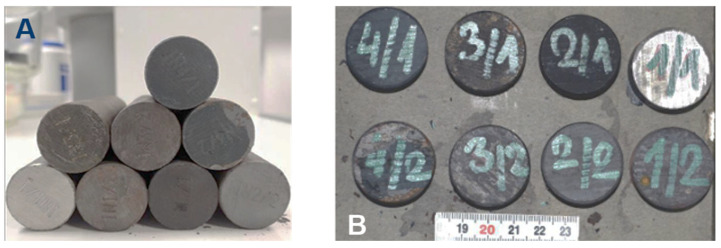
Steel cylindrical bars obtained from the manufacturer: (**A**) Side view, (**B**) Top view.

Steel cylindrical rods Ø 31 × 300 mm made from the steel 41CrMo4FD were obtained from Sidenor S.L., Spain, in the as-rolled state (see Figure 3) with a Young’s modulus (E) value of 210 GPa and Poisson’s ratio (ν) of 0.29. All these samples were heat-treated to develop different residual stresses by different heat treatment modes, and are used to evaluate the behaviour of the developed sensors to different magnitudes of stress.

To introduce different levels of internal stress, several heat treatment modes were performed based on austenitization at 880 °C and subsequent tempering at 520 °C. Different heat treatments were chosen: stress relieving, quenching into oil (OQ), quenching into water (WQ), Furnace cooling (FC) and air cooling (AC). The delivered rods were cut into two identical halves to get a set of just quenched and quenched and tempered pairs of samples. Parameters of the individual heat treatment modes of rods are stated in Table 2.

**Figure 4:**
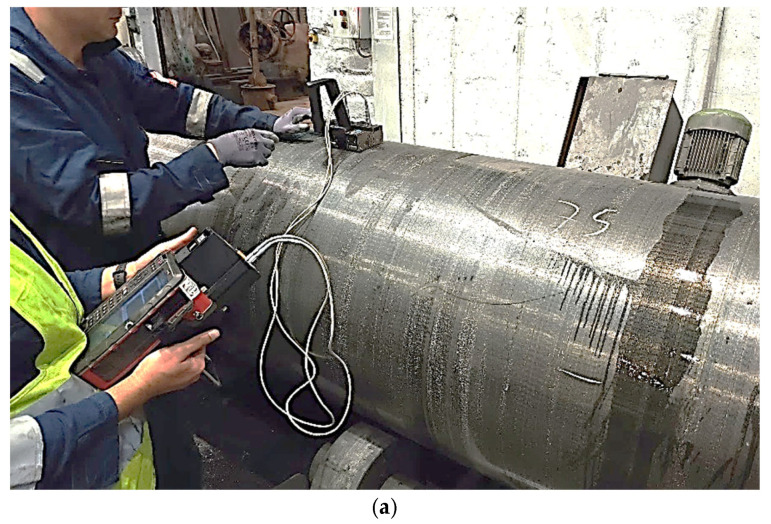

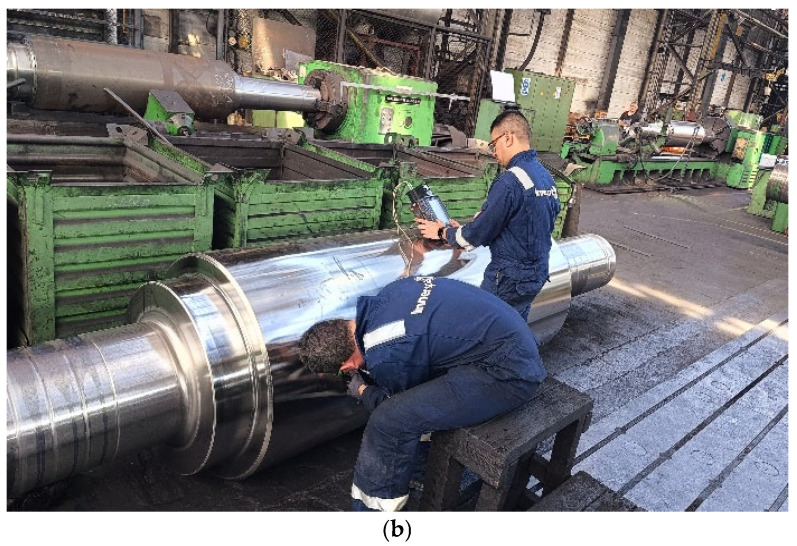
EMAT Inspection of steel rolls at Valji facility (**a**) before grinding, (**b**) after grinding.

At the Valji steel roll facility, Innerspec personnel have conducted extensive inspections on different steel rolls. The inspections involved measuring samples in both rough ground and polished conditions (as shown in Table 3). This comprehensive approach provides a thorough understanding of the material’s properties and potential defects across different processing stages and surface finishes. Figure 4 provides a glimpse into the performed continuous inspections, showcasing the capabilities of utilizing EMAT nonlinear ultrasonic technology in evaluating steel rolls.

## 4. Methodology Used in the Research

### 4.1. X-Ray Diffraction

XRD is a powerful technique for analyzing the structural properties of materials and can be particularly useful for measuring residual stress. XRD is a non-destructive analytical technique used to determine the crystal structure, phase composition, and other structural parameters of materials. When X-rays interact with a crystalline material, they are diffracted according to Bragg’s Law (Equation (3)):

(3) nλ=2d sinθ

where n is the order of reflection, λ is the wavelength of the incident X-rays, d is the interplanar spacing of the crystal planes, and Ѳ is the angle of incidence (Bragg angle). The resulting diffraction pattern provides information about the arrangement of atoms in the crystal lattice and can be used to analyze the material’s residual stresses. XRD can be employed to measure residual stress through a technique known as the sin^2^ψ method, which involves the following steps:

- Diffraction Peak Shifts:

Residual stress causes shifts in the diffraction peaks observed in an XRD pattern. By analyzing these shifts, one can infer the presence and magnitude of internal stresses. Specifically, the lattice parameters change in response to stress, leading to variations in the diffraction angles.

**Figure 5:**
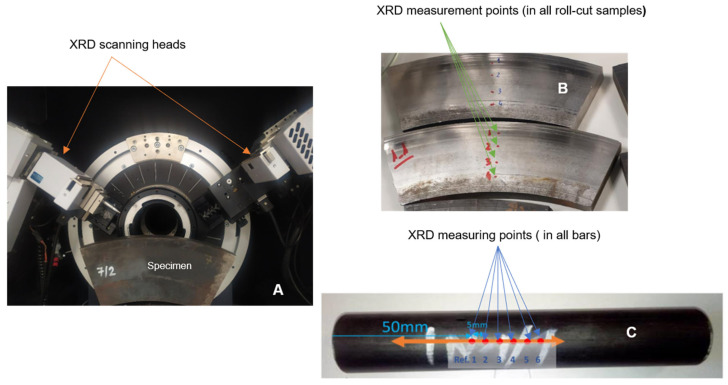
Smaller industrial roll and bar samples measured using XRD: (**A**) XRD machine, (**B**) XRD measuring points in cut-rolls, (**C**) XRD measuring points in bars.

- The Sin^2^ψ Method:

The sin^2^ψ method is commonly used to quantify residual stress. In this approach, XRD measurements are performed at various tilt angles (ψ) relative to the surface of the material. An exemplary Figure 5A shows the XRD analysis of the Valji 7/2 specimen using the sin^2^ψ method.

The procedure involves:

1. Sample Preparation: The sample is prepared with a known crystallographic orientation.
2. Diffraction Measurement: XRD scans are taken at different ψ angles. Each measurement corresponds to a different orientation of the sample relative to the X-ray beam.
3. Data Analysis: The shifts in diffraction peaks are plotted against sin^2^ψ to determine the stress-free lattice parameter (d_0_) and the residual stress in the material. The stress can be calculated using Equation (4):

(4) $$\sigma =\frac{E}{\left(1+\nu \right)}\frac{\left(d-d_{0}\right)}{\left(d_{0}\right)}$$

where σ is the residual stress, E is Young’s modulus of the material, ν is Poisson’s ratio, d is the measured lattice spacing, and d_0_ is the stress-free lattice spacing.

Figure 5B,C show the XRD setup for Valji and Sidenor samples, positioned parallel to the table. XRD sensing was conducted at four and six points (marked by green and blue arrows). As a pointwise method, XRD provides surface-level residual stress values. The tests were performed by the Tekniker Research Centre in the Basque region, Spain.

### 4.2. EMAT and Rayleigh Waves

**Figure 6:**
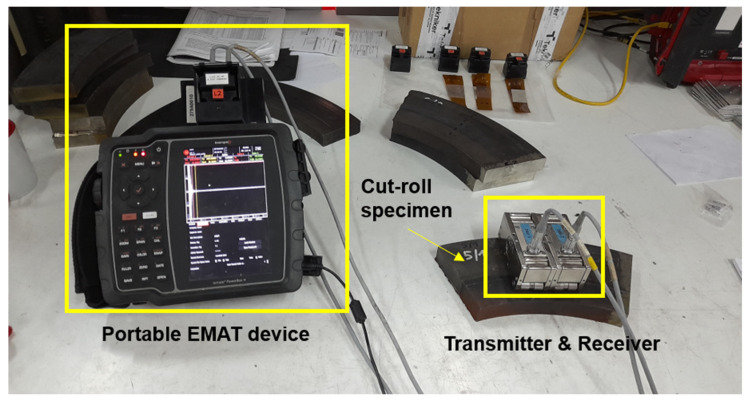
Innerspec Portable EMAT device with a lab specimen under investigation.

**Figure 7:**
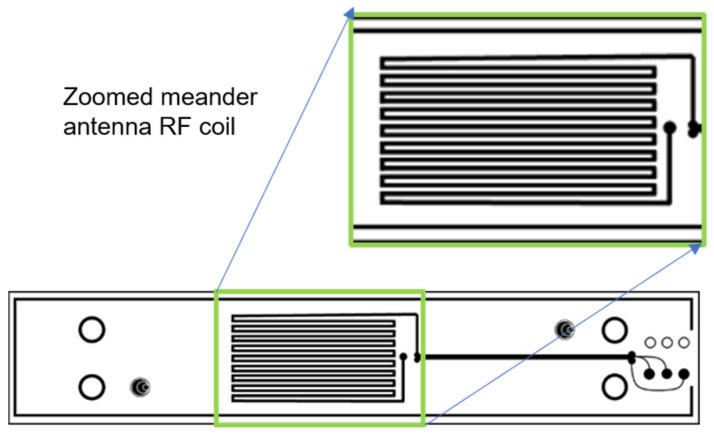
Schematic EMAT meander coil.

**Figure 8:**
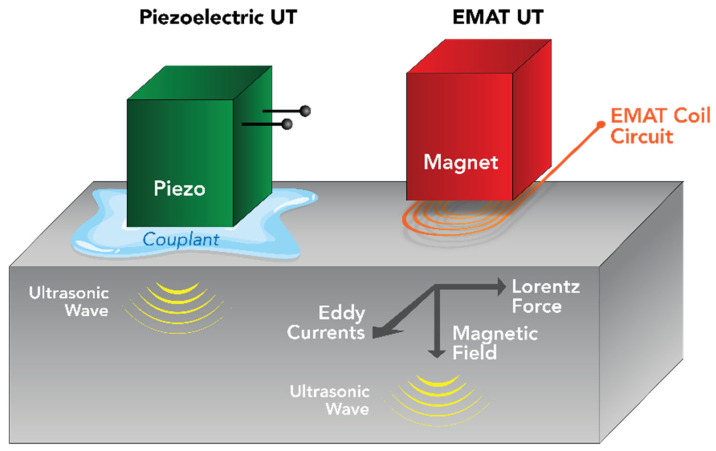
Explanation of EMAT technology vs. Piezo Ceramics [23].

The nonlinear ultrasonic study to monitor residual stresses of the obtained specimens was conducted using Rayleigh waves generated using an in-house EMAT device named Power Box H (Figure 6), which uses meander EMAT coils (Figure 7) to create such guided-surface waves in the solid objects. An Ultrasonic Testing (UT) technique called EMAT, for short, produces sound in the part being inspected rather than the transducer itself. An EMAT uses two interacting magnetic fields (Figure 8) to create ultrasonic waves inside a test object. Similar to an electric motor, a low-frequency or static field produced by magnets interacts with a relatively high-frequency field produced by electrical coils to produce a Lorentz force. An elastic wave is created when this disruption is transferred to the material’s lattice.

However, it is critical to recognize that the depth at which residual stress can be accurately measured using Rayleigh waves which is limited by their penetration depth, typically around 90–95% of the wavelength (as used in Equation (5)). Rayleigh waves are a type of surface acoustic wave that travel along the surface of a solid, causing elliptical particle motion and decaying with depth. Their sensitivity to surface and near-surface properties makes them particularly useful for probing residual stresses and other mechanical characteristics without damaging the material. This methodology has practical applications in materials science and engineering, allowing researchers to gain insights into the mechanical properties and behavior of a variety of materials. In this study, Rayleigh waves were employed as the primary method to assess stress levels in various samples (Table 1 and Table 2). The waves were generated using a meander coil of specific wavelength, and measurements were conducted in a pitch-catch mode [24] configuration, where a transmitter and receiver were positioned in a one-send, one-receive arrangement.

#### 4.2.1. Residual Stress Monitoring: Concept

**Figure 9:**
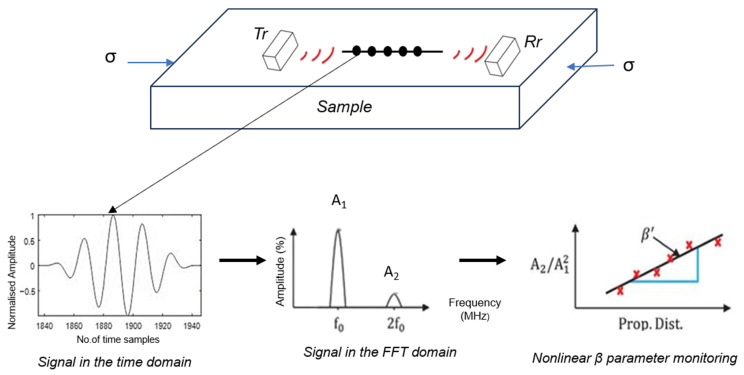
Exemplary residual stress monitoring using Rayleigh waves.

Residual stress within a material can be monitored by analyzing Rayleigh wave signals in the frequency domain. As these waves propagate through a material, their interaction with the stress can induce nonlinear effects, such as the generation of a second harmonic component at frequency (2f_0_). The top part of Figure 9 shows Rayleigh waves being introduced into the material, represented by a rectangular block with a transmitter and receiver placed parallel to each other. As these waves travel through the stressed region, nonlinear interactions cause the generation of a second harmonic, which can be detected and analyzed.

The lower left of Figure 9 shows the Rayleigh wave signal as a function of time. This signal exhibits variations in amplitude as it propagates through the material. To analyze this signal for stress monitoring, it is first pre-processed using a Hanning window to minimize spectral leakage before performing the FFT. The resulting amplitude spectrum, shown in the middle of Figure 9, clearly displays the presence of a fundamental frequency component at (f_0_) and a second harmonic component at (2f_0_). The amplitudes (A_1_) and (A_2_) corresponding to these two frequency components are extracted directly from the FFT magnitude spectrum as the peak values at (f_0_) and (2f_0_), respectively, by selecting the time signal’s main signal, which is chosen based on cutting the time domain signal to a specific range. To improve repeatability, the amplitudes are averaged over multiple acquisitions (4 times) and normalized with respect to the input excitation voltage, ensuring that the nonlinear parameter estimation is independent of instrumentation effects.

In addition, the influence of beam spreading and material attenuation, which disproportionately affect higher harmonic amplitudes, was carefully considered. Frequency-dependent attenuation coefficients (∝f_0_) and ∝2f_0_) were experimentally determined by measuring Rayleigh wave amplitudes at different propagation distances in the unstressed state. The measured amplitudes were then corrected using exponential compensation factors, A_corr_ = A exp(∝x), where (x) is the propagation distance. This ensures that the ratio (A_2_/A_1_^2^) reflects the intrinsic material nonlinearity rather than propagation losses.

The linear relationship depicted by the slope ‘β’ in Figure 9 indicates that, as the wave propagates through the material (Equation (2)), the second harmonic grows at a rate proportional to the stress-induced nonlinearity. This slope β serves as a quantitative measure of the residual stress within the material. By applying the above corrections and analyzing this relationship, one can reliably assess the distribution and magnitude of residual stresses along the propagation path of the Rayleigh wave.

In summary, residual stress monitoring using Rayleigh waves involves introducing a wave into the material, detecting the induced second harmonic due to nonlinear interactions with stress, applying appropriate windowing and attenuation compensation during signal processing, converting the wave signal from the time domain to the frequency domain, and analyzing the growth of the second harmonic with propagation distance to determine the nonlinear parameter β.

#### 4.2.2. Residual Stress Monitoring Process

**Figure 10:**
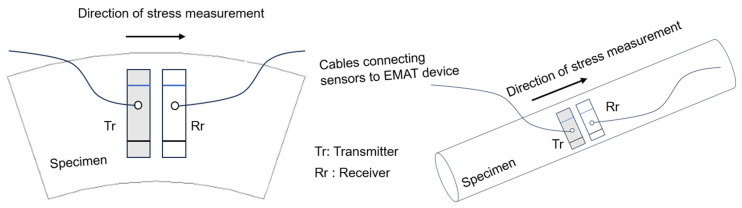
The direction of stress measurement in the lab specimens.

**Figure 11:**
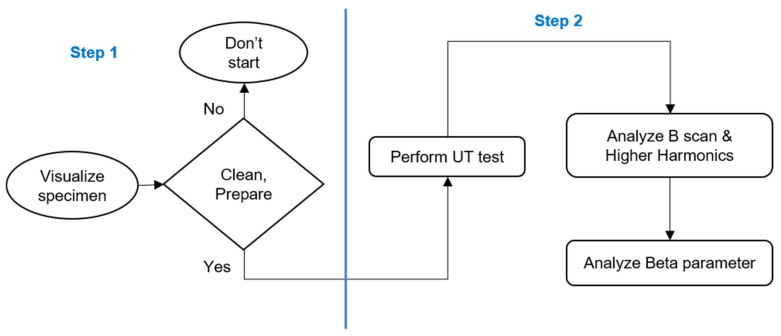
Flowchart of the process followed for both pointwise and continuous inspections.

The following methodology is used in inspecting the specimens (Figure 10), which is done using the ultrasonic nonlinear method. The process is explained as a flowchart in Figure 11, and it is elaborated in detail as follows:

Step 1: Visualization and initial inspection

The specimens were visually inspected and, if as-cast, lightly cleaned with a cloth to remove excess iron ore dust. This dual-purpose cleaning ensures stress measurements and protects the EMAT sensors, which contain magnets prone to attracting iron ore particles.

Step 2: UT-EMAT inspection: Lab and Industrial

Innerspec’s PBH equipment (Figure 6) generates Rayleigh waves per specified settings (Table 4). An in-house written script processes received Rayleigh wave signals into B-scan (group of Ascan) plots and conducts FFT analysis to detect frequencies. Residual stress, often signalled by higher FFT harmonics (Figure 1), is assessed by identifying frequencies A_1_ and A_2_ and applying Equation (2) to determine the non-linear β parameter along the specimen’s length. This analysis is optimal for NDT studies employing a pitch-catch sensor setup across the entire structure length.

A relevant aspect when positioning the sensor on the specimen is the direction of transmission of the signal. The signal travels between the transmitter and the receiver, and this direction determines the direction of the stress that is measured. In the case of the cut rolls and bars, this direction was perpendicular to the length of the pieces, as shown in Figure 10.

Figure 6 shows that the EMAT sensor, due to its larger size, is placed between points 2 and 3 on the sample (refer to Figure 5). Instead of measuring all four points as in the XRD analysis, an average of the XRD results from points 2 and 3 is used. This average is then compared with the EMAT results obtained from the same central region. The coercive force results, with sensors placed between points 2 and 3, are also compared in this way.

In pointwise studies, the inspections are performed at discrete points, meaning measurements are taken at specific moments or locations in the process. In contrast, the developed nonlinear ultrasonic analysis enables continuous inspection of the industrial specimen. Instead of stopping to measure at individual points, the analysis and monitoring occur in real time, providing constant assessment of the specimen. This continuous approach is particularly advantageous for high-volume or critical industrial operations, as it allows immediate detection of defects or variations without interrupting the workflow. While all steps from the flowchart are followed in the same sequence, they are adapted to accommodate the continuous inspection process.

**Figure 12:**
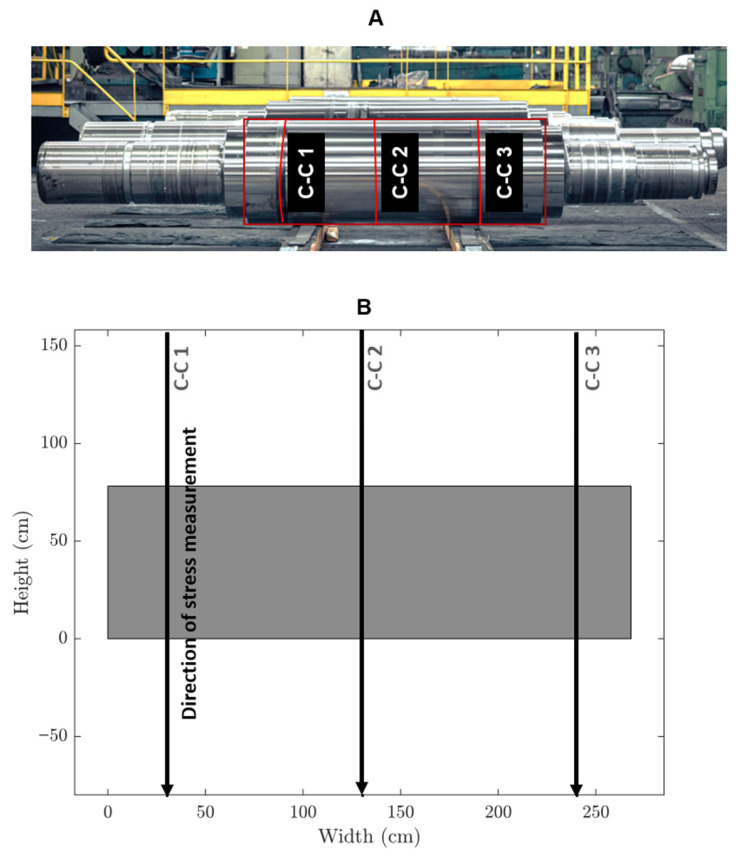
Continuous monitoring: (**A**) steel roll with column-wise centre (C-C) inspected sections, (**B**) the schematic version of it.

**Figure 13:**
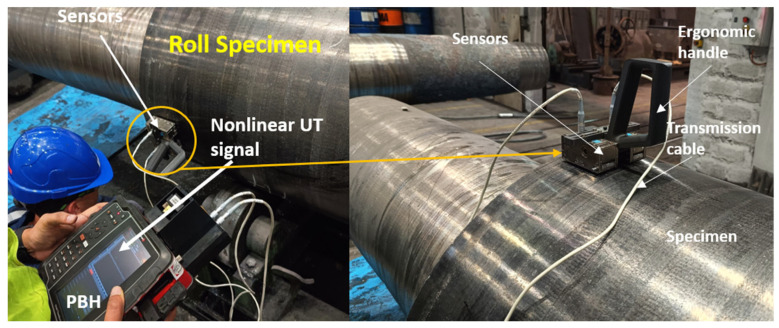
Portable EMAT device and pitch-catch sensors configuration at Valji facility.

Figure 12 shows an industrial roll specimen divided into three distinct columnar sections, labelled C-C1, C-C2, and C-C3. The EMAT analysis targets these columns, which are continuously monitored during inspection. Nonlinear ultrasonic analysis is performed specifically on these regions to measure the key parameter, β, in real time. Unlike discrete pointwise measurements, this approach evaluates β continuously along the length of the central columns by moving the sensors from top to bottom. This continuous measurement enables more detailed and immediate detection of variations or defects in the material properties of the specimen. The roll figure, along with its schematic, visually highlights the analyzed regions and emphasizes the segmentation of the columns for nonlinear analysis. Figure 13 illustrates the industrial inspection setup at the Valji d.o.o. roll manufacturing facility using EMAT sensors.

### 4.3. Coercive Force

Coercivity refers to the measure of a material’s ability to withstand an external magnetic field without becoming demagnetized. It is a critical property in magnetic materials and is defined as the intensity of the applied magnetic field required to reduce the magnetization of a material to zero after it has been saturated.

- Magnetic Hysteresis:

Coercivity is part of the magnetic hysteresis loop, which illustrates how a material’s magnetization responds to an applied magnetic field. The hysteresis loop demonstrates the relationship between the magnetic field strength and the resulting magnetization, showing how a material retains its magnetization after the external field is removed.

- Coercivity Value:

High Coercivity: Materials with high coercivity can maintain their magnetization in the presence of external magnetic fields. These materials are often used in permanent magnets and magnetic storage media (e.g., hard drives). Materials with low coercivity are easily demagnetized and are typically used in applications where the magnetic properties need to change frequently, such as in transformer cores and magnetic shielding.

**Figure 14:**
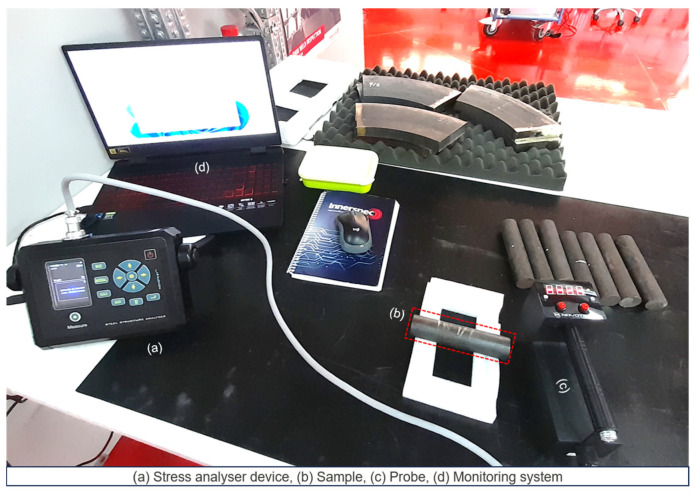
Coercive force analysis setup.

- Measurement:

Coercivity is measured by subjecting a sample to a magnetic field until it is saturated, then reducing the field to zero and measuring the field required to reduce the magnetization to zero. This value is obtained from the hysteresis loop as the coercive field (Hc). Figure 14 shows the coercivity analyser equipment obtained from Novotest Ltd., which helps to correlate coercivity with the relative residual stress in the material.

Coercivity and residual stress are related in the context of magnetic materials because residual stress can influence the magnetic properties of a material, including its coercivity. By measuring the hysteresis loop of a material, including its coercivity, before and after applying residual stress, one can assess how residual stress influences coercivity.

## 5. Results and Discussions

### 5.1. Dispersion Calculations

A 0.06-inch wavelength (λ) meander EMAT coil was selected for residual stress monitoring after comparing smaller (0.032-inch) and larger (0.08- and 0.1-inch) coils. This coil offered an ideal penetration depth of approximately ±1.5 mm while providing a higher signal-to-noise ratio than the alternatives, making it most effective for capturing near-surface stress variations. The corresponding Rayleigh wave carrier frequency is determined using Equation (5).

F = (0.92 × V_p_/(λ)) (5)

where V_p_ is the steel’s phase velocity, which is taken as 3.13 mm/µs, λ is the wavelength of the EMAT coil and is taken as 0.06 inches (1.524 mm). The coefficient 0.92 comes from the theoretical relationship between the Vp and λ for a free surface in an isotropic solid. The value 0.92 accounts for the slight difference between the Rayleigh wave velocity and the shear wave velocity of the material. This formulation is widely used in ultrasonic NDT literature, for example, see Rose [26] and Viktorov [27].

**Figure 15:**
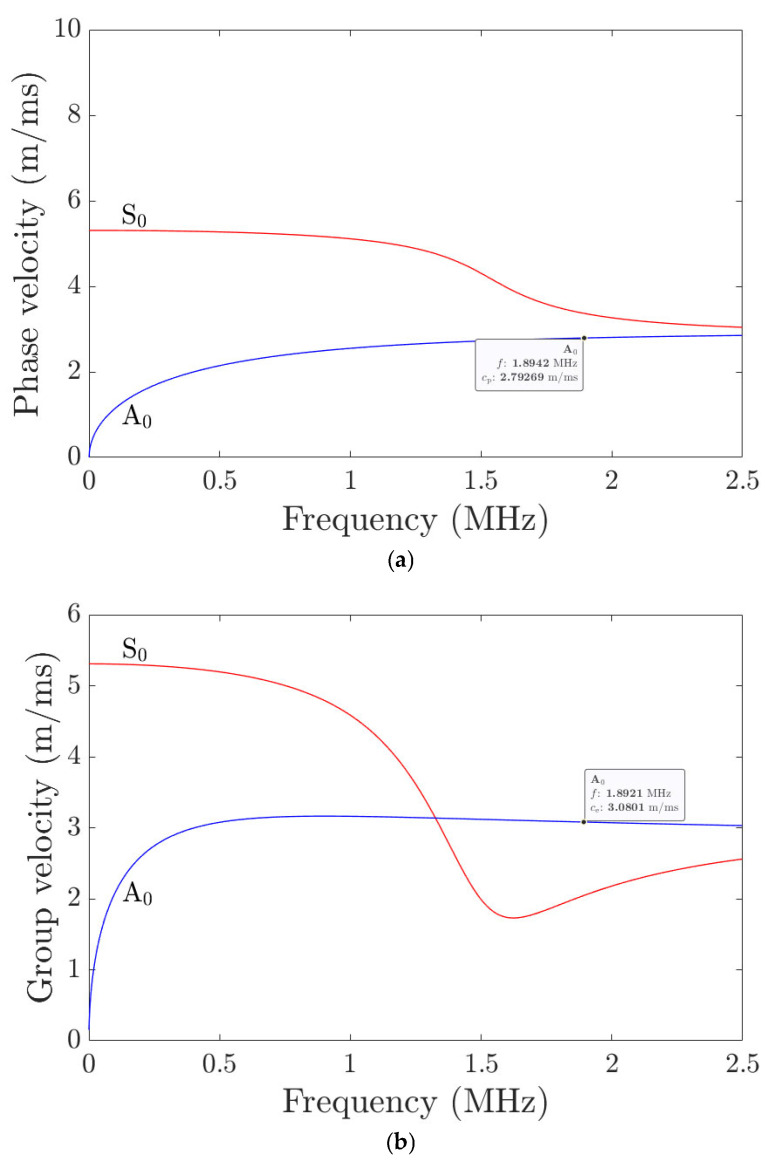

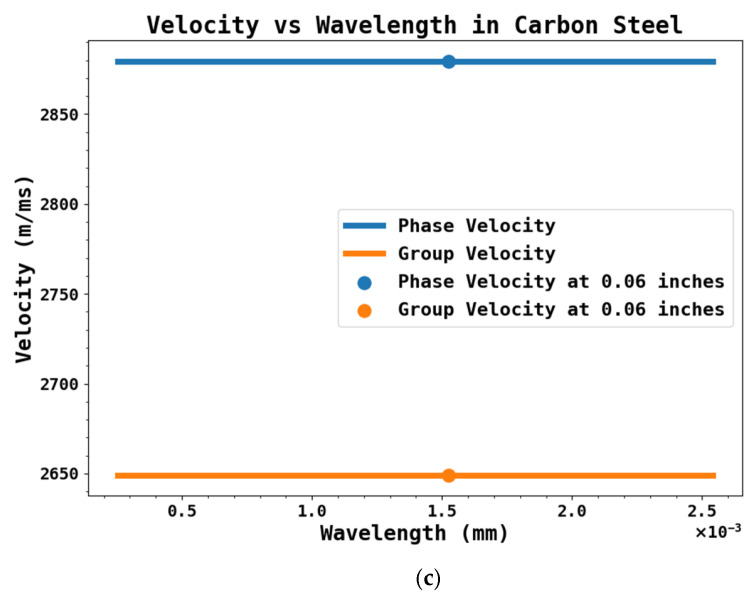
Dispersion numerical calculations: Rayleigh waves in Carbon steel (**a**) phase velocity, (**b**) group velocity, (**c**) analytical calculations.

Thus, 1.889 MHz is the obtained carrier frequency (F) for the measurements where Rayleigh waves are generated for the specified wavelength based on Equation (5). In the initial part, the analytical calculations were carried out with the developed equations to obtain the analytical phase and group velocity (V_g_) values for Rayleigh surface waves, as shown in Table 5. The numerical (obtained using a Dispersion calculator [28]) and analytical results are shown in Figure 15. The calculations are made for a thickness of 1.524 mm (wavelength 0.06 inch), and Rayleigh waves are primarily sensitive over a depth on the order of the wavelength. The comparative calculations are shown in Table 6.

**Figure 16:**
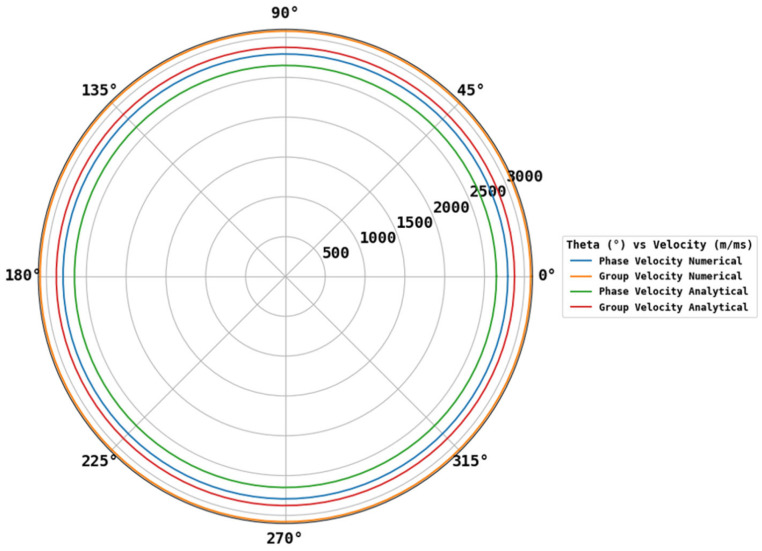
Polar plot obtained from the results of numerical and analytical calculations.

The phase velocity calculations obtained from the numerical and analytical methods were also identical, as shown in Figure 16 polar plot plotted all along the orientations. The difference between the analytical and numerical group velocity was around 200 m/ms, which we assume is due to the change in the material property used in numerical calculations.

### 5.2. Analysis of Valji Lab Samples

The XRD results from four different points were collected, as detailed in Table 7 from Table 1 specimens. The data encompasses multiple stress (σ) measurements at each point, labelled as 1/1, 2/1, 2/2, 3/1, 3/2, 4/1, and 4/2, and are recorded in MPa. These measurements reveal the stress variations at different points across the sample (Figure 5), highlighting both positive and negative stress values. To streamline the analysis and present a more comprehensive overview, the individual measurements from points 2 and 3 were averaged. This averaging process aimed to condense the data into a single representative value, providing a comparative understanding of the stress distribution at the centre of the samples. The averaged results are shown in Table 8, offering a simplified depiction of the stress behaviour as observed in the XRD measurements.

In the EMAT study, the A_1_ and A_2_ parameters (Table 9) were obtained using nonlinear technology, which is particularly effective in capturing the nonlinearity of the material under examination. The parameter β was also determined (Equation (2)) in this analysis. The sensors were strategically placed at the centre of the sample due to their large size (Figure 6), ensuring that the measurements accurately reflected the central region where stress concentrations and material behaviour are most critical.

Table 10 represents the coercive force results obtained using the Novotest coercive force analyzer. These results were measured in A/cm for various samples. Similar to the previous approach, a large sensor was placed at the centre of the sample (Figure 14) to ensure accurate measurement, given the significant size of the material under investigation.

**Figure 17:**
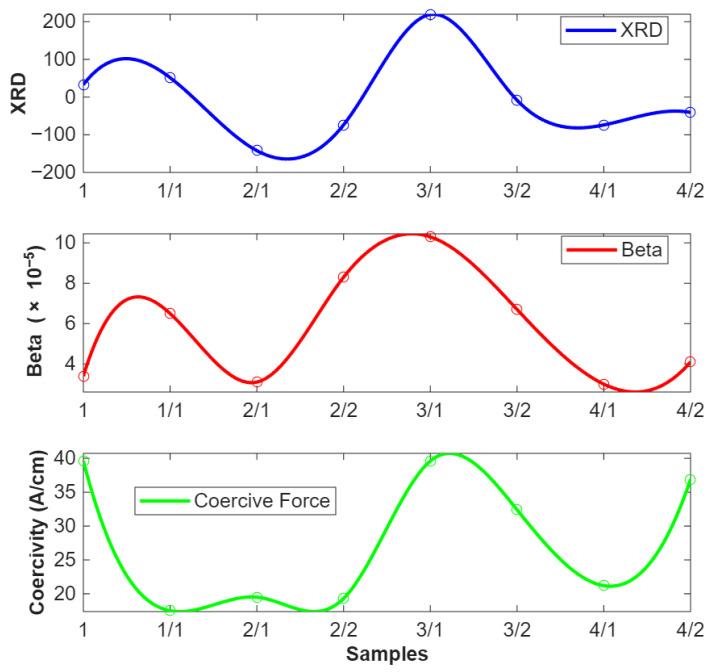
Roll samples: Comparative graphs of XRD, coercivity, and β values.

Figure 17 features three-line plots that display measurements of XRD, β, and Coercive force across eight steel roll-cut samples. The top panel shows XRD data, which indicates residual stress, with positive values representing tensile stress and negative values representing compressive stress. For instance, sample 3/1 has a peak in XRD, indicating significant tensile stress, while sample 2/1 shows a trough, suggesting compressive stress.

The middle panel displays the β plot, which reflects variations in crystallite size and is also related to relative residual stress. Higher β values, such as those seen in sample 3/1, are consistent with greater residual stress, echoing the trends observed in the XRD data. In the bottom panel, the Coercive force plot measures the material’s resistance to demagnetization. Variations in coercivity can be associated with changes in residual stress, with samples showing lower coercivity (like 1/1 and 2/1) potentially having less residual stress, while higher coercivity in samples such as 3/1 indicates more significant residual stress. Overall, the plots show a similar trend across the measurements, with XRD, β, and Coercivity all reflecting variations in residual stress.

### 5.3. Analysis of Sidenor Lab Samples

The XRD results from six different points (Figure 5C) were collected, as detailed in Table 11 of Table 2 Specimens. The data encompasses multiple stress (σ) measurements at each point, labelled as 1/1, 1/2, 2/1, 2/2, 3/1, 3/2, 4/1, and 4/2 and are recorded in MPa. These measurements reveal the stress variations at different points across the sample, highlighting both positive and negative stress values. To streamline the analysis and present a more comprehensive overview, the individual measurements from points 2 and 3 were averaged from the XRD results. This averaging process aimed to condense the data into a single representative value, providing a comparative understanding of the stress distribution at the centre of the samples. The averaged results are shown in Table 11. The β and coercive force values obtained at point 3 from all the samples are also added to the comparative table.

**Figure 18:**
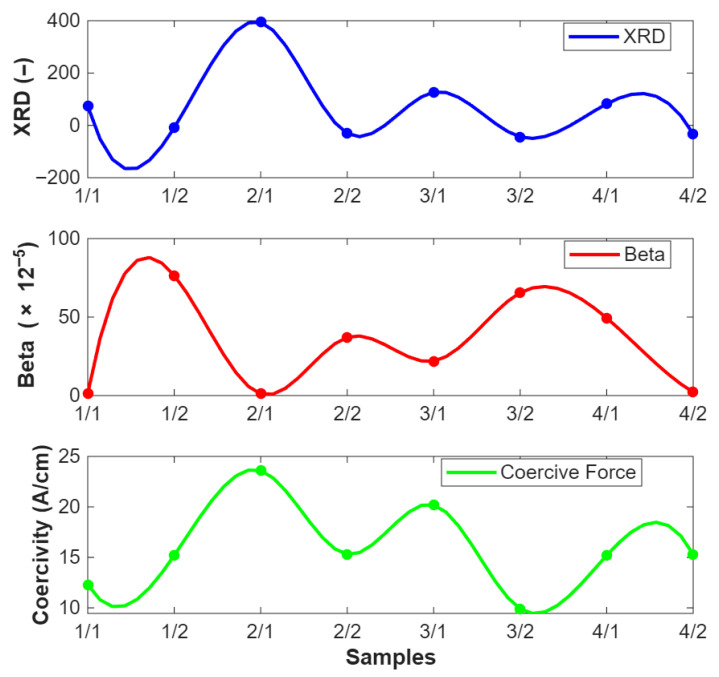
Bar samples: Comparative graphs of XRD, Coercivity, and β values.

The top graph in Figure 18 shows XRD measurements across various samples. The values vary significantly across samples, indicating differences in the lattice strain and hence the residual stress. The middle graph represents the nonlinear ultrasonic parameter β. The trend here also shows variation across samples, with some positive trend to the XRD data, suggesting that this method is sensitive to the same types of internal changes. The bottom graph shows Coercivity measurements, which indicate how the material’s magnetic properties are affected by internal stresses. The trend in this graph appears to be a good fit with the other two methods to some extent, especially in samples where the XRD and β values are high.

All three methods, XRD, β, and Coercivity, are sensitive to changes in the material’s microstructure, which are often induced by residual stresses. This similarity suggests that each method is capable of detecting the internal stress state of the material from different perspectives. While there is some positive trend between the methods (e.g., high XRD values often coincide with high β or Coercive force values), the differences also highlight that each method might be more sensitive to different types of microstructural changes. This variability is crucial for gaining a complete picture of the residual stress distribution and its effects on the material.

### 5.4. Continuous Monitoring of Industrial Rolls

**Figure 19:**
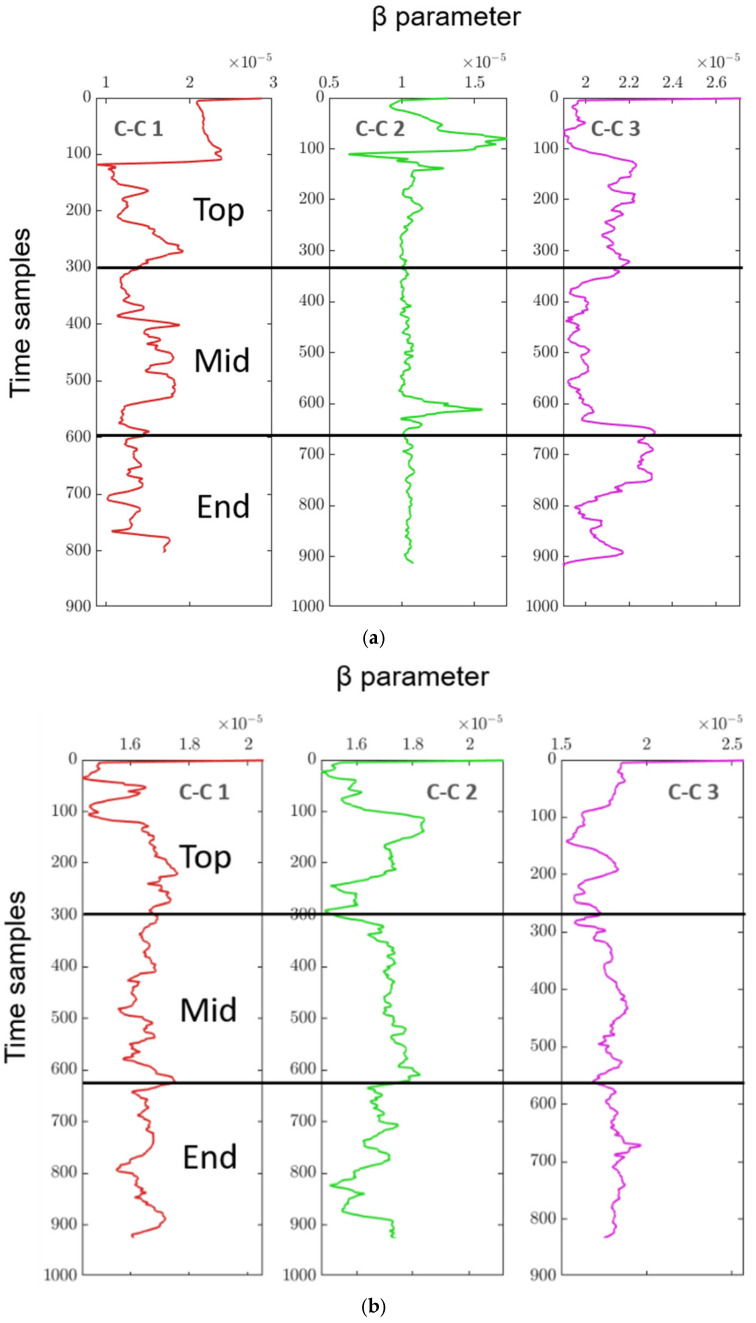
Continuous β monitoring results of the steel roll (**a**) before grinding and (**b**) after grinding.

In Figure 19, the continuous monitoring results of the β parameter are shown for three columns (C-C 1, C-C 2, and C-C 3 as shown in Figure 12) at different positions (Top, mid, and End) before and after a grinding process. Figure 19a shows the β values before grinding for the top, mid, and end sections of the columns of a steel roll. The β parameter fluctuates continuously for each column and section, indicating the nonlinear behaviour of the specimen material. For each of the three columns (C-C 1, C-C 2, C-C 3), the time samples on the *y*-axis represent the progression of continuous inspection over time, while the *x*-axis shows the measured β parameter values.

Figure 19b displays the β parameter measurements after grinding of the same steel roll. Similar to Figure 19a, the results for the top, mid, and end positions are presented for each column. The fluctuations of the β parameter still exist but are notably smoother in some regions compared to before grinding, suggesting a change in material properties due to the grinding process. In both figures, the analysis was performed continuously across the columns, as explained in Figure 12, to monitor the specimen’s condition in real time. Further observation of the probable tension and compression in the measured whole specimen is explained in Table 12 (T: probable region of tension, C: probable region of compression). The data helps highlight the changes in material behaviour both pre- and post-grinding, showing how the nonlinear characteristics evolve through continuous β parameter evaluation across different sections of the columns.

Table 12 presents an analysis of steel rolls before and after grinding, comparing different surface states (from the foundry and after finishing) across three positions on the roll: Top, Mid, and End. The focus is on the variation in β values, which seem to indicate stress states related to T and C in these regions.

Steel roll before grinding:
The steel rolls are in a heat-treated state and roughly ground. Across the different sections of the sample (C-C 1, C-C 2, and C-C 3), similar patterns are observed:

- Top: There is a noticeable fluctuation in β values, suggesting both T and C regions. This indicates that the surface is under varied mechanical stress, where certain areas are being stretched (tension) while others are compressed.
- Mid: The β values show fluctuation, indicating a mix of T and C states. This combined stress profile may result from uneven cooling or handling during heat treatment, leading to residual stresses in the steel.
- End: Like the mid-section, the β values indicate a combination of T and C regions.

The variation in β values before grinding suggests that the steel rolls, while heat-treated, have not reached a balanced or uniform stress state. This could impact the material’s structural integrity if left untreated, possibly causing issues during later processing steps.

Steel roll after grinding:

After the rolls are finished and measured again before machining, there is a noticeable improvement in some areas:

- Top: The β values continue to fluctuate, but there is still a suggestion of combined T and C regions, indicating that the grinding process has not fully relieved the stress in this part.
- Mid: The β values remain fluctuating, showing a persistent mix of T and C states, similar to before grinding.
- End: Interestingly, the β values are now more stable, suggesting a more balanced stress state after grinding. This is especially notable in the C-C 2 section of the sample, where the end section exhibits stable values, pointing to a significant improvement in reducing residual stresses.

Overall, grinding does affect the stress profile of the steel rolls, particularly at the ends, where a more balanced stress state is achieved post-grinding. This might be due to the removal of surface irregularities or defects that were present in the rough state. Despite this, the top and mid-sections continue to show combined T and C regions, which suggests that grinding alone may not be sufficient to eliminate all residual stresses in these areas. The persistence of fluctuating β values in the top and mid-regions even after grinding may indicate the need for additional stress-relief processes, such as further heat treatment or controlled machining. While the grinding helps in balancing the stress state, particularly at the ends of the rolls, tension and compression remain a challenge in the top and mid-sections. These areas may require further attention to achieve a uniform stress distribution, which is critical for the roll’s performance and durability during its service life.

### 5.5. Statistical Correlation and Limitations

**Figure 20:**
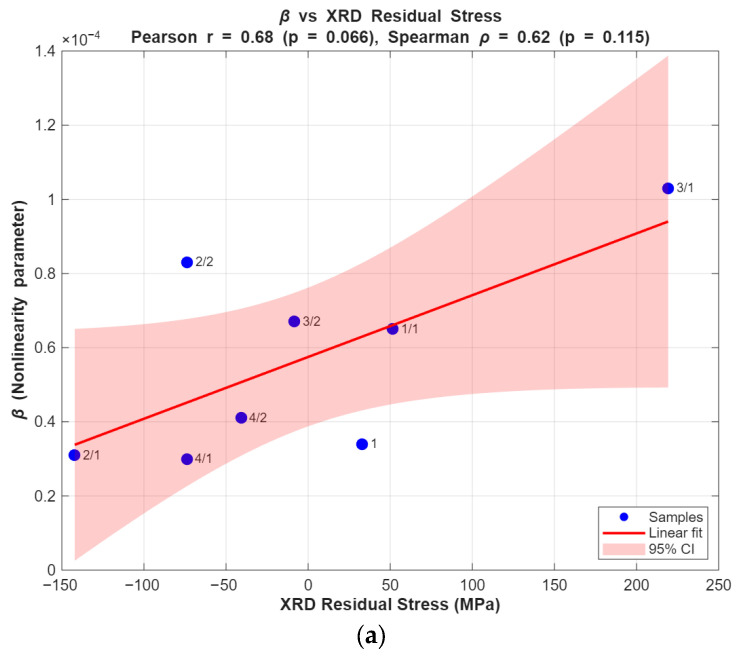

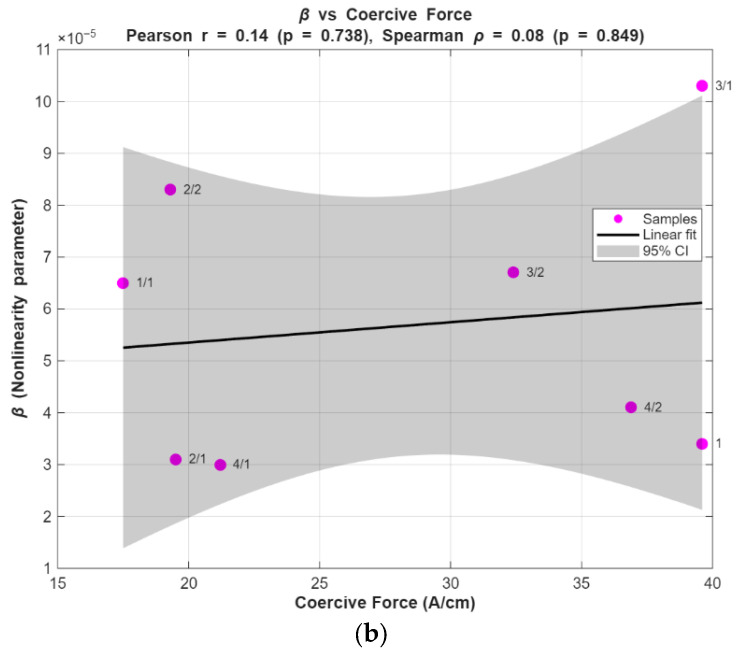
Quantitative correlation analysis of rolls (**a**) XRD (vs.) β, (**b**) Coercive force (vs.) β.

To quantify the relationship between the β and the reference measurements, Pearson and Spearman correlation coefficients were calculated for the Valji laboratory samples (n = 8). The Pearson correlation between β and XRD-measured residual stress is r = 0.68 (*p* = 0.066), and the Spearman rank correlation is ρ = 0.62 (*p* = 0.10), indicating a moderate positive association between β and XRD stress values. By contrast, the correlation between β and coercive force is weak (Pearson r = 0.14, *p* = 0.74; Spearman ρ = 0.08, *p* = 0.84). The plots obtained using the linear regression model are shown in Figure 20 with a broader confidence level (CL) of 95%.

**Figure 21:**
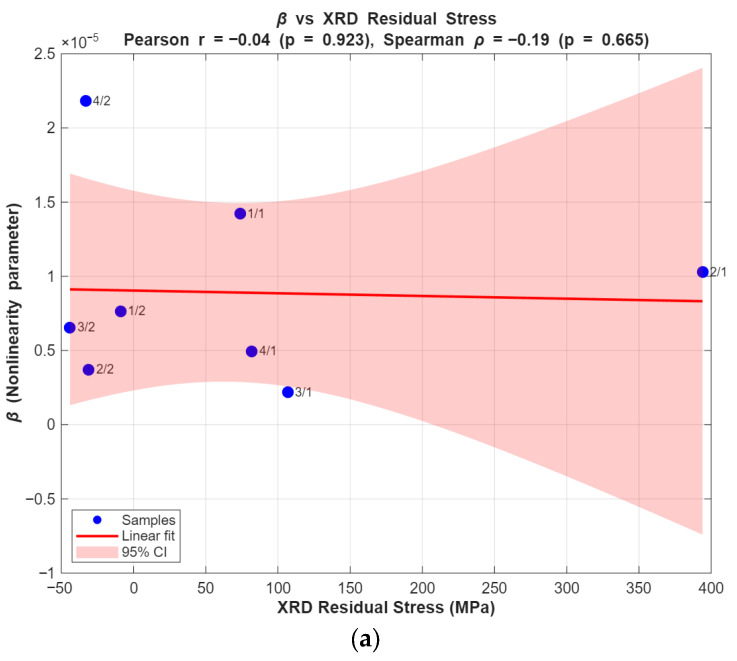

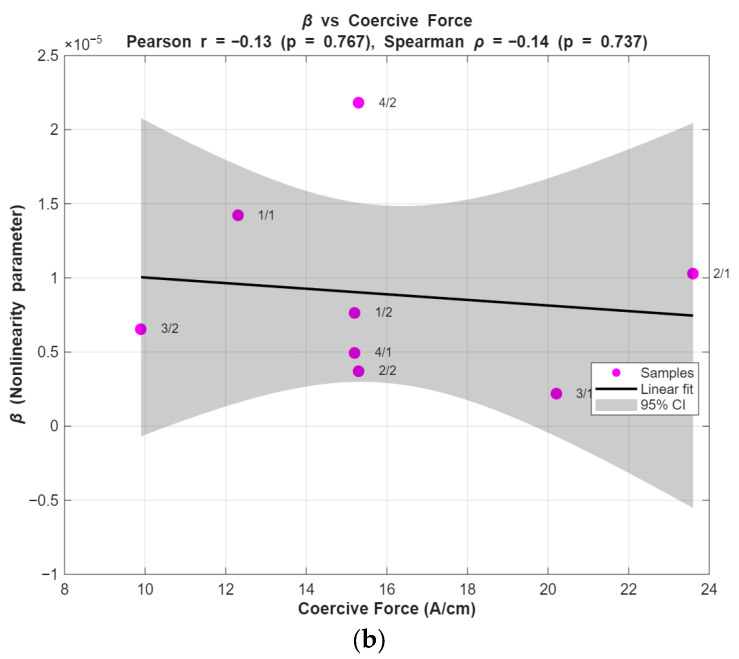
Quantitative correlation analysis of bars (**a**) XRD (vs.) β, (**b**) Coercive force (vs.) β.

To evaluate the above relationship for the available steel bars, eight samples (n = 8) similar to the above-mentioned parameters were used. The correlation between β and XRD-measured residual stress was negligible, with Pearson r = −0.04 (*p* = 0.923) and Spearman ρ = −0.19 (*p* = 0.651), indicating no meaningful association. Similarly, the correlation between β and coercive force was weak, with Pearson r = 0.14 (*p* = 0.74) and Spearman ρ = 0.08 (*p* = 0.84). Linear regression plots with 95% confidence intervals are shown in Figure 21.

These results indicate that the relationship between β and XRD is a trend rather than a robust statistically significant correlation with the current dataset. The lack of statistical significance at the 0.05 level is likely due to the small sample size (n = 8) and the presence of mixed tensile/compressive states in the dataset, which increases variance.

Important limitations that may explain deviations between methods are: (1) measurement depth mismatch EMAT Rayleigh-wave sensitivity extends to roughly 1.5 mm beneath the surface (meander coil used), whereas XRD probes only the near-surface (order of ~10 µm); (2) different physical contrast mechanisms β quantifies acoustic nonlinearity related to microstructural features and dislocations, XRD quantifies lattice strain, and coercivity depends on magnetic domain behavior and surface finish; (3) surface condition and roughness can affect coercivity and XRD more strongly than EMAT; (4) mixed tension/compression regions within a single sample can reduce linearity of correlation; and (5) limited sample size reduces statistical power.

### 5.6. Prototype Development

**Figure 22:**
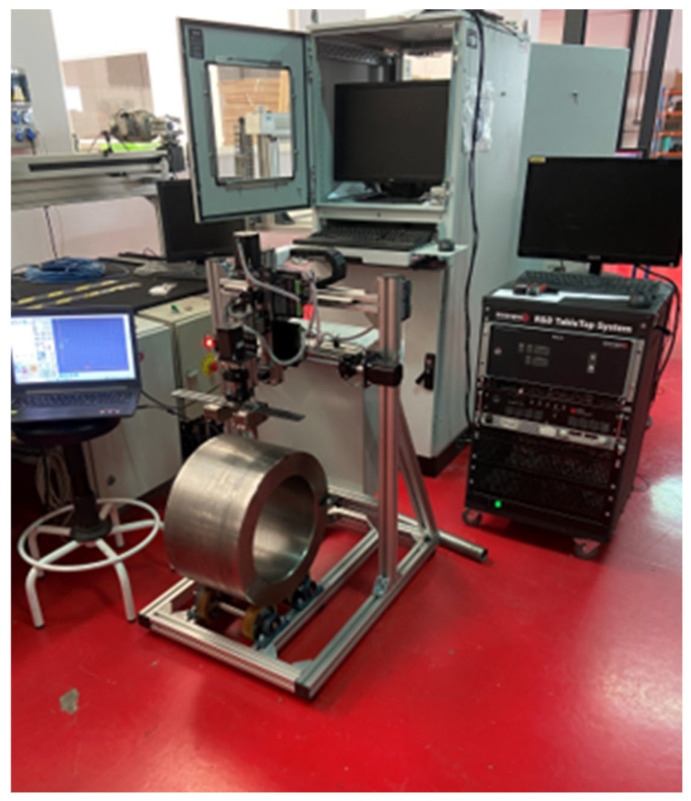
Prototype developed for the continuous monitoring of steel structures.

The research project led to the development of a specialized prototype system aimed at performing continuous nonlinear ultrasonic testing on roll samples, as illustrated in Figure 22. The system consists of a mechanical setup that positions the roll sample for precise ultrasonic wave transmission, utilizing an EMAT to generate and receive Rayleigh waves. This continuous testing approach allows for real-time monitoring of residual stresses within the material, enabling immediate data collection and analysis. The prototype, which integrates various components such as a computer interface, data acquisition systems, and imaging equipment, provides instant results on the stress distribution across the roll.

This capability is particularly valuable for industrial applications, where quick assessments of structural integrity can lead to more informed decision-making and improved maintenance strategies for large steel structures. The continuous nature of the testing, coupled with immediate results, enhances the effectiveness of the method for long-term monitoring and offers a significant advantage over traditional techniques that may require longer processing times.

## 6. Conclusions

The study provides a comprehensive analysis of residual stress in steel rolls and bars, employing nonlinear ultrasonics as the primary investigative technique. This method was compared with traditional approaches such as XRD and coercivity measurements. The study meticulously details the methodology, including the preparation of steel samples, the specific heat treatments applied to induce varying levels of residual stress, and the subsequent analysis using different techniques. The research demonstrates that nonlinear ultrasonics, particularly when used in conjunction with Rayleigh waves and EMATs, offer significant advantages in terms of sensitivity and depth of analysis, especially for subsurface residual stresses that are often challenging to detect with conventional methods.

The Key findings of this research include the following:

- This research validates nonlinear ultrasonics, using EMAT, as an effective tool for monitoring stress distributions in steel components.
- The technique’s capability to detect subsurface residual stress with a positive trend compared to XRD and coercivity measurements is particularly significant. This suggests that nonlinear ultrasonics can often serve as a good and rapid alternative for residual stress analysis in industrial steel components, rather than merely a complementary method.
- Unlike traditional methods, which are slow and limited to pointwise measurements, EMAT-based nonlinear ultrasonics enables fast and continuous monitoring.
- Through comprehensive comparison, the study shows that nonlinear ultrasonics not only detects stress variations with greater precision but also provides deeper insights into the material’s internal condition, which other methods may overlook.
- In both steel roll and bar samples, EMAT effectively identified variations in residual stress caused by different heat treatments, demonstrating pattern recognition.
- The continuous monitoring capability of EMAT technology makes it a highly valuable tool for real-time residual stress analysis in industrial environments, minimizing downtime and improving the structural integrity of essential steel components.
- The continuous monitoring was performed at a 15 km/hr speed, which was crucial in rapid analysis.
- Before grinding, the steel rolls exhibit fluctuating β values, indicating a mix of T and C across all sections, with an unbalanced stress profile. After grinding, the end sections show a more balanced stress state, but the top and mid-sections still have combined T and C, suggesting residual stress remains. Grinding improves stress distribution, particularly at the ends, but further treatment may be needed for uniformity.
- By significantly enhancing the accuracy of residual stress measurements, nonlinear ultrasonics contributes to a deeper understanding of material behaviour, ultimately increasing the reliability and lifespan of steel components used in industrial settings.
- The research also developed a prototype system for continuous nonlinear ultrasonic monitoring, enabling real-time, efficient analysis of residual stresses in industrial steel components. This prototype’s integration with EMAT technology ensures immediate results, enhancing decision-making and maintenance strategies for critical steel components.

In conclusion, this research study successfully positions nonlinear ultrasonics as a valuable and potentially superior tool for residual stress analysis in steel. The findings suggest that its broader adoption in industrial settings could lead to more effective monitoring and maintenance of steel components, ultimately leading to improved safety and performance in critical applications.

Further work involves expanding the application of this technology to other materials and more complex structural geometries, refining the prototype system for even greater accuracy and usability in industrial environments. Additionally, long-term studies on the effectiveness of continuous monitoring in real-world conditions will be crucial for validating its potential for widespread use in various sectors.

## Figures and Tables

**Table 1:** Heat-treatment parameters for the roll segments.

Sample	2/1	2/2	3/1	3/2	4/1	4/2
Quenching	950 °C/1 h/water		950 °C/1 h/oil		950 °C/1 h/air	
Tempering	-	550 °C/2 h	-	550 °C/2 h	-	550 °C/2 h

**Table 2:** Heat-treatment parameters for the bar segments [A-R as received state].

Sample	1/1	1/2	2/1	2/2	3/1	3/2	4/1	4/2
Quenching	-	-	880 °C/1 h/OQ		880 °C/1 h/OQ		880 °C/1 h/OQ	
Tempering	A-R	720 °C/3 h/FC	no	520 °C/2 h/AC	no	520 °C/2 h/AC	no	520 °C/2 h/AC

**Table 3:** Industrial specimens inspected.

S. No	Dept.	Cast State	Heat Treated	Rough Grinding	Polished Grinding	Dimensions (mm)
1	Foundry	-	Yes	Yes	-	Φ732 × 2000
2	Finished	-	-	-	Yes	Φ930 × 2230

**Table 4:** EMAT device settings used in the research study.

S. No	Requirements	Parameters
1	Wave mode used	Rayleigh waves
2	Coil type	0.06-inch meander
3	Central frequency used	1889 kHz
4	Window function	Sine
5	No. of Sine cycles	5
6	Bandpass filter used	500 kHz–8000 kHz
7	Sampling Frequency	25 MHz
8	FFT window length	2048 points
9	No. of signal Averages	4
10	Signal to Noise (SNR) Threshold	5

**Table 5:** Analytical calculations.

Shear Velocity (V_s_)	Wavelength	V_p_ [0.92 × Vs]	V_g_ [0.92 × Vp]
3.13 mm/s	0.06 inches	2879.6 m/ms	2649.2 m/ms

**Table 6:** Comparative calculations.

Numerical		Analytical	
Phase velocity (m/ms)	Group velocity (m/ms)	Phase velocity (m/ms)	Group velocity (m/ms)
2792	3080	2649.2	2876.6

**Table 7:** XRD values obtained from Valji samples.

Points	1 σ (MPa)	1/1 σ (MPa)	2/1 σ (MPa)	2/2 σ (MPa)	3/1 σ (MPa)	3/2 σ (MPa)	4/1 σ (MPa)	4/2 σ (MPa)
1	66.7	47.5	−323.5	−259.2	193.1	61.4	−279.3	40.5
2	−15.3	5.4	−165	−101.6	137.3	8	−150.6	−20
3	81.2	97.7	−119.3	−45.8	301.2	−24.7	2.7	−61.6
4	149.1	218.1	−248.9	−154.7	56.1	−82.1	−238.4	−128.8

**Table 8:** Average values of the points 2–3 from Table 7.

Samples	XRD @ Points 2 and 3 (MPa)
1	32.95
1/1	51.55
2/1	−142.15
2/2	−73.7
3/1	219.25
3/2	−8.35
4/1	−73.95
4/2	−40.8

**Table 9:** Nonlinear analysis using EMAT device on Valji samples.

Samples	A_1_ (−)	A_2_ (−)	β (−)
1	439.6308604	6.501823898	0.000034
1/1	409.778622	10.98296949	0.000065
2/1	641.0616833	12.92907824	0.000031
2/2	335.9046824	9.385312151	0.000083
3/1	343.5509415	12.19394964	0.000103
3/2	606.8060754	24.72110057	0.000067
4/1	673.0498371	13.50488959	0.00003
4/2	711.0209904	20.78660503	0.000041

**Table 10:** Coercivity results from the samples.

Samples	Coercive Force (A/cm)
1	39.6
1/1	17.5
2/1	19.5
2/2	19.3
3/1	39.6
3/2	32.4
4/1	21.2
4/2	36.9

**Table 11:** Comparison plot table values from the XRD, β and coercivity.

Samples	XRD @ Points 2 and 3 (Average) MPa	β (−)	Coercive Force (A/cm)
1/1	74	1.4204 × 10^−5^	12.3
1/2	−9	7.6548 × 10^−6^	15.2
2/1	394	1.0275 × 10^−5^	23.6
2/2	−31	3.7103 × 10^−6^	15.3
3/1	107	2.1906 × 10^−6^	20.2
3/2	−44	6.5498 × 10^−6^	9.9
4/1	82	4.9434 × 10^−6^	15.2
4/2	−33	2.1803 × 10^−5^	15.3

**Table 12:** Summary of the results from before and after grinding rolls.

Roll Before Grinding	Top	Mid	End
C-C 1: from the foundry in a heat-treated state, the surface was roughly ground.	Increasing and decreasing β values (high fluctuation) suggest T and C regions.	Fluctuating β values indicate combined T and C states.	Fluctuating βvalues indicate combined T and C states.
C-C 2: from the foundry in a heat-treated state, the surface was roughly ground.	Increasing and decreasing β values suggest T and C regions.	Stable or fluctuating β values indicate a balanced stress state.	Stable β values indicate a balanced stress state.
C-C 3: from the foundry in a heat-treated state, the surface was roughly ground.	Increasing and decreasing β values suggest T and C regions.	Fluctuating β values indicate combined T and C states.	Fluctuating β values indicate combined T and C states.
Roll after grinding	Top	Mid	End
C-C 1: from the finished state and measured before machining.	Increasing and decreasing β values suggest T and C regions.	Fluctuating β values indicate combined T and C states.	Stable or fluctuating β values indicate a balanced stress state.
C-C 2: from the finished state and measured before machining.	Increasing and decreasing β values suggest T and C regions.	Fluctuating β values indicate combined T and C states.	Fluctuating β values indicate combined T and C states.
C-C 3: from the finished state and measured before machining.	Increasing and decreasing β values suggest T and C regions.	Fluctuating β values indicate combined T and C states.	Fluctuating β values indicate combined T and C states.

## Data Availability

The datasets, code supporting the findings of this study are available upon reasonable request to promote reproducibility and further research in the field of assistive technologies. The source code and documentation can be provided to researchers upon contacting the corresponding author.
